# Neuralgia.—Case of Miss E. R. L.

**Published:** 1848-07

**Authors:** J. Lee

**Affiliations:** Camden, S. C.


### ARTICLE IV.

### *Neuralgia. Case of Miss E. R. L.* By J. Lee, M. D., of Camden, S. C.

Miss L. spent the winter of 1836-'7 in Mobile and New Orleans; prolonged her residence until June, '37. Returning to South Carolina she was much exposed; several times wet; took severe cold; was attacked with neuralgic pains in the face; referred principally to the dens sapientiæ, inferior maxilla, right side. On examination I found two cavities not deep; I excavated and filled them; pain continued a week or ten days; I extracted the tooth; it came away easily; the tooth was not considered the cause of the pain, but it was crowded and could be well spared. The pain in the jaw increased. It was treated through the general system, and locally, by stimulants, galvanism, emollients, and as it manifested a periodicity of pain from bad to worse, it was treated with quinine, &c., &c. Nothing left undone from which hopes of benefit could be derived.

From November to January, 1838, scarce a night's rest was obtained; the whole system seemed to be giving way under intense suffering; the pain commenced at the right central incisor, and extended back to the angle of the jaw; the middle and upper branch of the nerve were only occasionally painful. At this stage of the disease she earnestly solicited the extraction of the teeth; this, of course, was refused. I proposed the examination of the first molar of that side which I had plugged with gold in 1832; removing the filling, I found the bottom of the cavity clean and white. I then drilled into the pulp, causing but little additional pain; the pulp was perfectly healthy. One-eighth grain of arsenic was placed in the cavity and covered with wax. At the expiration of two hours the pain began to diminish. That night she slept well. I renewed the arsenic, putting in half a grain and covered it with a tin filling. At the expiration of ten days I took out the tin, most of the arsenic had disappeared. The capsule of the tooth was very tender, being inflamed, as I suppose, from the absorption of the arsenic; this soreness continued more than a month. Three months have now elapsed since the operation. The neuralgic pain has not returned; the tooth has no sensibility. At present it has a tin filling.

